# Influence of Hesperidin on Systemic Immunity of Rats Following an Intensive Training and Exhausting Exercise

**DOI:** 10.3390/nu12051291

**Published:** 2020-05-01

**Authors:** Patricia Ruiz-Iglesias, Sheila Estruel-Amades, Mariona Camps-Bossacoma, Malén Massot-Cladera, Àngels Franch, Francisco J. Pérez-Cano, Margarida Castell

**Affiliations:** 1 Secció de Fisiologia, Departament de Bioquímica i Fisiologia, Facultat de Farmàcia i Ciències de l’Alimentació, Universitat de Barcelona (UB), 08028 Barcelona, Spain; patriciaruiz@ub.edu (P.R.-I.); sheilaestruel@ub.edu (S.E.-A.); marionacamps@ub.edu (M.C.-B.); malen.massot@ub.edu (M.M.-C.); angelsfranch@ub.edu (À.F.); 2 Institut de Recerca en Nutrició i Seguretat Alimentària (INSA-UB), UB, 08921 Santa Coloma de Gramenet, Spain

**Keywords:** blood, cytokines, exercise, exhaustion, lymphocytes, macrophages, NK cells, phagocytosis, spleen, thymus

## Abstract

Intensive training and exhausting exercise can disrupt innate and acquired immunity. The flavanone hesperidin has shown immunomodulatory properties in physiological and some pathological conditions, and positive effects on exercise-induced oxidative stress. Nevertheless, it remains uncertain whether it also prevents exhausting exercise-induced immune alterations. The aim of this study was to establish the effect of oral hesperidin supplementation on the systemic immune system in rats following an intensive training and exhausting exercise. For this purpose, female Wistar rats were randomized into an intensive training group or a sedentary group. Intensive training was induced by running in a treadmill 5 days per week (including two exhausting tests) for five weeks. Throughout the training period, 200 mg/kg of hesperidin or vehicle was administered by oral gavage three times per week. At the end, blood, thymus, spleen and macrophages were collected before, immediately after and 24 h after an additional final exhaustion test. Hesperidin supplementation enhanced natural killer cell cytotoxicity and the proportion of phagocytic monocytes, attenuated the secretion of cytokines by stimulated macrophages, prevented the leukocytosis induced by exhaustion and increased the proportion of T helper cells in the thymus, blood and spleen. These results suggest that hesperidin can prevent exhausting exercise-induced immune alterations.

## 1. Introduction

Nowadays, there is no doubt about the benefits of regular physical activity for health and in the prevention of several diseases. Sedentary life is a risk factor for cardiovascular disease and other chronic diseases, such as type 2 diabetes, cancer, osteoporosis, obesity, hypertension and depression [1,2]. Physical activity stimulates the body’s cardiorespiratory, musculoskeletal and metabolic systems, making them more efficient. It is important to point out that the immune system functionality is also influenced by physical exercise. In comparison with sedentary behavior, it is well established that regular or moderate physical activity improves the immune system defensive function [3]. However, its functionality can be reduced when the exercise performance is extreme or excessive. In fact, acute vigorous exercise alters almost all blood immune cells influencing their functional capacity [4,5,6,7]. As an example of detrimental effects of excessive physical activity, it has been reported that elite endurance athletes during periods of intensive training have a higher frequency of upper respiratory tract infections [4,8], similarly to the effects on non-elite runners following a marathon [9]. The damaging outcomes of physical exercise depend on its bout and load and these are still controversial [10]. Some data demonstrate that high training loads in athletes induce physiological alterations including, among others, impaired motor coordination, a decrease in muscle strength, a decrease in maximal oxygen uptake and endurance capacity [8], certain inflammatory status and oxidative stress [11], which all together can eventually result in decreased performance. We have recently demonstrated that intensive training and exhausting exercise in rats impairs innate immunity [6], in particular the phagocytic and the natural killer (NK) cytotoxic activities and macrophage cytokine secretion. This excessive exercise also has an impact on acquired immunity, inducing changes in the lymphocyte distribution and functionality [7].

In the field of nutritional support, there is interest in interventions that help to increase exercise performance, to reduce fatigue and to counteract the detrimental effects associated with extreme physical activity. For periods of intense physical activity, ensuring adequate energy, carbohydrate and protein intake as well as avoiding deficiencies of micronutrients are key strategies to avoid detrimental effects on the body and maintain immune health [12]. Among micronutrients, evidence is accumulating that supplements with flavonoids as well as other polyphenols can be useful for athletes. Flavonoids are products of plants secondary metabolism and are consumed through vegetables in the diet. In general, they are poorly absorbed in the human small intestine, reaching the colon where microbiota metabolizes them into products that can be absorbed and exert bioactive effects [13]. Flavonoids, as they are polyphenols, are able to improve antioxidant defenses and they are suggested to counteract strenuous exercise-associated oxidative stress and inflammation. In this context, it has been reported that consumption of quercetin for one or two weeks by untrained volunteers results in higher performance in bike-cycling [14] and treadmill training [15]. Likewise, consumption of a Montmorency cherry juice concentrate, containing high levels of flavonoids, improves the recovery of isometric muscle strength after intensive exercise in well-trained athletes [16]. 

Within the flavonoid family, citrus fruits mainly contain flavanones such as hesperidin. Besides its antioxidant capacity [17], hesperidin has demonstrated beneficial biological activities in systemic and intestinal immunity [18,19]. With regard to physical activity, it has been demonstrated that supplementation with a citrus flavonoid extract for 4 weeks improves cycling time-trial performance in trained male athletes [20]. In addition, rats receiving a hesperidin supplementation for 4 weeks and submitted to swimming, improved the biochemical profile and antioxidant biomarkers [21]. Moreover, hesperidin supplementation for 5 weeks in intensively trained rats prevented oxidative stress induced by intensive and exhausting exercise and improved exercise performance [22]. Based on this background, the current study aimed to ascertain the influence of hesperidin supplementation in the systemic immune system functionality in rats following an intensive training and exhausting exercise.

## 2. Materials and Methods

### 2.1. Animals

Sixty-four three-week-old female Wistar rats (Envigo, Huntingdon, United Kingdom) were maintained in polycarbonate cages containing bedding of large fibrous particles (Souralit 1035, Bobadeb S.L., Santo Domingo de la Calzada, Spain) (4 rats per cage) under controlled conditions (temperature and humidity, in a 12/12 h light/dark cycle) in the animal facilities of the Faculty of Biology at the University of Barcelona. Female rats were chosen because previous studies showed that this gender had better adaptability and higher performance than male rats [6]. Animal procedure was approved by the Ethical Committee for Animal Experimentation of the University of Barcelona and the Catalonia Government (CEEA/UB ref. 464/16 and DAAM 9257, respectively), in full compliance with national legislation following the European Union (EU)-Directive 2010/63/EU for the protection of animals used for scientific purposes. The sample size required (*N* = 8/group) was determined by the Appraising Project Office’s program from the Universidad Miguel Hernández de Elche (Spain), using the leukocyte total number for this calculation.

### 2.2. Diet and Hesperidin Supplementation

Water and food (Teklad Global 14% Protein Rodent Maintenance Diet, Teklad, Madison, WI, USA) were provided ad libitum throughout the study. Body weight (BW) and food intake were also monitored. Food efficiency was calculated by dividing the total BW gain in a cage by the total food intake of the same cage.

A half of animals, including both runner and sedentary groups, received a supplement of hesperidin (HealthTech BioActives, Murcia, Spain) administered by oral gavage three times per week at a dose of 200 mg/kg BW in 0.5% carboxymethylcellulose solution, prepared daily. This dosage was established due to its immunomodulatory effects evidenced in previous studies [18,19]. Non-supplemented rats received the same volume of the vehicle.

### 2.3. Exercise Training Program and Samples Collection

After a 7-day acclimation period, exercise training began by using two specialized treadmills for rodents: a LE8700 treadmill (Panlab, Harvard, USA) and an Exer3/6 treadmill (Columbus, OH, USA). The exercise training program has been previously described [6,7]. Briefly, all the 64 rats were firstly adapted to the treadmill for 10 days by increasing time and speed. At the end of this adaptation period, rats were randomly divided into four groups: non-supplemented runner animals (RUN, *n* = 24), hesperidin-supplemented runner animals (H-RUN, *n* = 24), non-supplemented sedentary animals (SED, *n* = 8) and hesperidin-supplemented sedentary animals (H-SED, *n* = 8). Thereafter, hesperidin supplementation began and both RUN group and H-RUN group were submitted to a 5-day period with increasing speed and duration followed by a 5 weeks’ intensive training. In each week, rats carried out an exhaustion test every Monday and Friday, whereas on Tuesday, Wednesday and Thursday rats ran for a limited and increasing time according to the maximum speed achieved in the previous Monday’s exhaustion test. Throughout the training program, SED and H-SED were exposed to the same conditions of isolation in a turned-off treadmill for the same period as runner rats. After each running session, runner rats received a solution of 50% condensed milk (100 µL/100 g BW) as a positive reward to reinforce their running. Sedentary animals also received this solution.

At the end of the 5-week training program, each runner group (RUN and H-RUN) was distributed into three subgroups, each one with a similar average in the ability to run, and two of these three groups carried out an additional final exhaustion test. The three subgroups allow the immune function assessment at different time points. For RUN and H-RUN animals, the groups were: Trained rats (T and H-T groups, *n* = 8 each one), which were euthanized 24 h after a regular training session, Trained with Exhaustion (TE and H-TE groups, *n* = 8 each one), which were euthanized immediately after carrying out an additional final exhaustion test, and Trained and after 24 h of Exhaustion (TE24 and H-TE24 groups, *n* = 8 each one), which were euthanized 24 h after the additional final exhaustion test. 

Sedentary rats (both SED and H-SED groups) were euthanized randomly distributed over the 3 consecutive days. Animals were anesthetized with ketamine (90 mg/kg, Merial Laboratories S.A., Barcelona, Spain) and xylazine (10 mg/kg, Bayer A.G., Leverkusen, Germany). Peritoneal macrophages, blood from heart, thymus, and spleen were immediately collected and processed. Peritoneal macrophages allowed the determination of cytokine secretion after stimulation. Blood was used to determine leukocyte differential counts by an automated hematology analyzer (Spincell, MonLab Laboratories, Barcelona). Other blood samples were used for characterizing lymphocyte composition and for establishing the phagocytic activity. Lymphocytes from thymus and spleen were isolated and used for characterizing lymphocyte composition. From spleen lymphocytes, the quantification of the proliferative response and the assessment of NK cell cytotoxic activity were also carried out. 

### 2.4. Lymphocyte Composition in Blood, Thymus and Spleen

Thymus and spleen lymphocytes were isolated by smashing tissues in a sterile mesh cell strainer (40 µm) as previously described [7,23]. Splenocyte suspensions and blood samples were submitted to erythrocyte lysis. Afterwards, lymphocyte subsets were determined by mouse anti-rat CD161b, CD45RA, CD8α, CD8β, CD4, TCRαβ or TCRγδ antibodies (BD Biosciences, San Diego, CA, USA) conjugated to fluorescein isothiocyanate (FITC), phycoerythrin, peridininchlorophylla protein, allophycocyanin or brilliant violet 421, as described previously [7,23]. Blood B cell proportion was approached by considering those lymphocytes negative for TCRαβ, TCRγδ and CD161b antibodies. In the thymus, the subsets considered were those bearing TCRαβ, which indicate mature thymocytes, and those expressing CD4 and/or CD8, which allow their classification in double negative (DN) cells (the most immature), double positive (DP) cells (the following state of maturation), and single positive cells, i.e., CD4+CD8- cells and CD4-CD8+ cells (the most mature thymocytes).

Data were acquired with a Gallios™ Cytometer (Beckman Coulter, Miami, FL, USA) in the Flow Cytometry Unit of the Scientific and Technological Centers of the UB (CCiTUB) and analyzed with FlowJo v.10 software (Tree Star, Inc., Ashland, OR, USA). The percentage of positive cells in the lymphocyte population selected was established according to forward-scatter characteristics (FSC) and side-scatter characteristics (SSC) or in a particular lymphocyte population.

### 2.5. Phagocytic Activity

Blood monocyte and granulocyte phagocytic function was assessed by flow cytometry analysis using the Phagotest^TM^ kit (Glycotope, Biotechnology GmbH, Heidelberg, Germany) in accordance with the manufacturer’s instructions, as previously reported [6]. Data were acquired using Gallios™ Cytometer (CCiTUB) and the analysis was performed with FlowJo v.10 software. Monocyte and granulocyte subsets were selected according to their FSC/SSC. The percentage of phagocytic monocytes and granulocytes was quantified by means of the proportion of FITC+ cells, whereas their phagocytic activity was measured through mean fluorescence intensity (MFI). 

### 2.6. Macrophage Cytokine Production

Peritoneal macrophages were collected by injecting cold phosphate-buffered saline (PBS) into the peritoneal cavity as previously reported [22]. Macrophages were suspended in cold Roswell Park Memorial Institute (RPMI) medium, supplemented with 10% heat-inactivated fetal bovine serum (FBS), 100 IU/mL streptomycin-penicillin and 2 mM L-glutamine, plated into 12-well plates (10^6^ cells/mL) and incubated for 2 h. After removing non-attached cells, macrophages were stimulated with 100 ng/mL lipopolysaccharide (LPS); non-stimulated macrophages were used as control. Supernatants were collected after overnight incubation. Interferon (IFN)-γ, interleukin (IL)-1β, IL-6, IL-10 and tumor necrosis factor (TNF)-α were quantified by using ProcartaPlex^®^ Multiplex Immunoassay (Affymetrix, eBioscience, San Diego, CA, USA), as previously described [21]. Data were acquired by Luminex MAGPIX analyzer (Luminex^®^) in the CCiTUB and analyzed with ProcartaPlex^®^ Analyst (Thermo Fisher Scientific, S.L.U, Barcelona, Spain). 

### 2.7. Natural Killer (NK) Cell Cytotoxic Activity 

The cytotoxic activity of spleen NK cells was quantified by the NKTEST^TM^ kit (Glycotope, Biotechnology GmbH, Heidelberg, Germany) following the manufacturer’s protocol as previously reported [6]. Data were acquired by the Gallios™ Cytometer (CCiTUB) and the analysis was performed with FlowJo v.10 software. Spontaneous cell death (without effector cells) was considered as control. Results from the individual cytotoxic activity were calculated according to the total NK activity and the percentage of NK cells of each sample.

### 2.8. Spleen Lymphocyte Proliferation

Spleen lymphocytes (10^5^ cells/well) were stimulated for 48 h, with either concanavalin A (ConA, 5 µg/mL, Sigma-Aldrich, Madrid, Spain) or pokeweed mitogen (PWM) (10 µg/mL, Sigma-Aldrich) to stimulate T and B cells, respectively. Assay was performed in quadruplicate and nonstimulated cells were maintained under the same conditions. Proliferative cells were quantified using a BrdU Cell Proliferation Assay kit (MerckMillipore, Darmstadt, Germany), as previously described [7,24]. Results are expressed as the percentage of cell proliferation increase after ConA and PWM stimulation with respect to unstimulated cells.

### 2.9. Plasma Cortisol Concentration

Plasma cortisol concentration was measured using DetectX^®^ Cortisol enzyme-linked immunosorbent assay (ELISA, Arbor Assays, MI, USA) in accordance with the manufacturer’s instructions. Absorbance was measured on a microplate photometer (Labsystems Multiskan, Helsinki, Finland) and data were interpolated by Ascent v.2.6 software (Thermo Fisher Scientific) according to the respective standard.

### 2.10. Statistical Analysis

Analysis of the data was carried out using IBM Social Sciences Software Program (SPSS, version 22.0, Chicago, IL, USA). The equality and normality of the data were tested by Levene’s and Shapiro−Wilk’s test, respectively. A two-way analysis of variance (ANOVA) test was applied and, if significant differences were detected, Tukey’s post hoc test was performed. A Kruskal−Wallis test was used when results were neither equally nor normally distributed, followed by Mann−Whitney U test in the case of significant difference among groups. To compare variables during the study (e.g., changes in maximum time supported in the exhaustion tests), a repeated-measures ANOVA was applied followed by Student’s *t*-test. Significant differences were considered when p≤0.05, except regarding repeated comparisons (Student’s *t*-test), when p value was corrected (Bonferroni correction), dividing it by the number of applied tests.

## 3. Results

### 3.1. Effect of Hesperidin Supplementation on Food Efficiency and Training Performance

**Figure 1:**
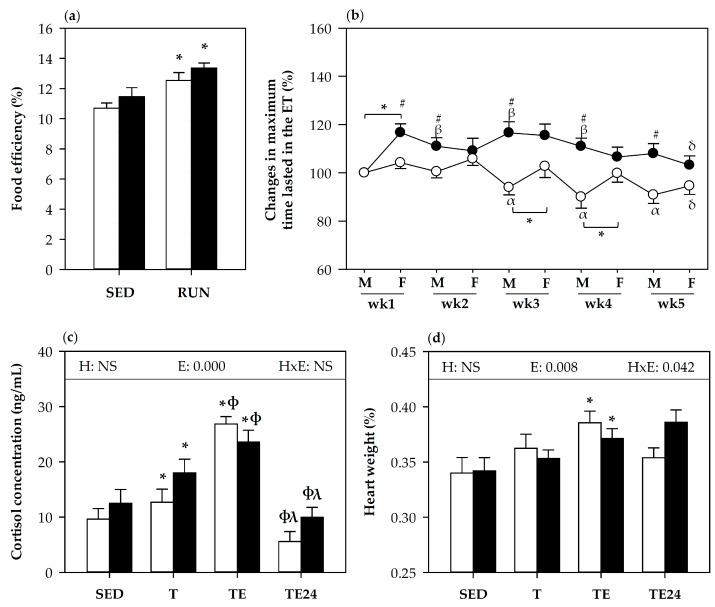
Food efficiency during the study (**a**), changes in maximum time supported in the exhaustion tests performed throughout the study (percentage with respect to the first exhaustion test) (**b**), cortisol concentration (**c**), and relative heart weight (**d**) at the end of the study. ET = exhaustion test; F = Friday; M = Monday; RUN = runner animals; SED = sedentary animals; T = trained groups; TE = T groups with an additional exhaustion test; TE24 = TE groups 24 h after the exhaustion test; wk = week. NS = no statistically significant differences detected. The non-supplemented group is represented by white symbols (○) and bars, and the hesperidin-supplemented group by black symbols (●) and bars. Data are expressed as mean ± standard error of the mean (SEM, *n* = 3-8 for **a**, *n* = 24 for **b**, *n* = 8 for **c** and **d**). Statistical difference in (**a**,**c**,**d**), the inset table shows two-way analysis of variance (ANOVA) results when applied (H, hesperidin; E, exercise, H–E, interaction between hesperidin and exercise): * *p* < 0.05 vs. SED group; ϕ *p* < 0.05 vs. T group; λ *p* < 0.05 vs. TE group. Statistical difference in (**b**): * significant differences between consecutive ETs for both non-supplemented and the hesperidin-supplemented groups (*p* < 0.05); α significant differences in the non-supplemented group vs. the first and the second Monday ET (*p* < 0.05); β significant differences in the hesperidin-supplemented group vs. the first Monday ET (*p* < 0.05) and δ significant differences in both non-supplemented and hesperidin-supplemented groups vs. the first Friday ET (repeated-measures ANOVA followed by paired Student’s *t* test); ^#^ significant differences between non-supplemented and the counterpart hesperidin-supplemented group (*p* < 0.005, Student’s *t*-test).

Body weight gain per rat (and the total per cage), and food consumption per cage were monitored throughout the study, allowing the calculation of the food efficiency. Both non-supplemented and hesperidin-supplemented runner animals showed higher food efficiency than the corresponding sedentary group and there were no significant effects due to hesperidin supplementation (Figure 1a). 

Figure 1b summarizes the changes in maximum time achieved in each exhausting test performed every Monday and Friday throughout the study considering the first exhaustion test as 100%. The mean time supported in the first exhaustion test was 23.83 ± 0.51 min and 22.39 ± 0.58 min (mean ± SEM) for non-supplemented and hesperidin-supplemented animals, respectively. Throughout the study and in general, rats receiving hesperidin ran for longer periods in the exhaustion tests than non-supplemented rats. The hesperidin-supplemented group improved performance for the first 4 weeks (each subsequent Monday ran for longer time than the first Monday) and did not show the Monday’s decreases in performance undergone by non-supplemented animals. Nevertheless, the improvement in physical performance due to hesperidin was not observed in the exhaustion tests carried out on the last three Fridays. At the end of the study, in the additional final exhaustion test, the maximum time supported by both hesperidin and non-hesperidin supplemented groups was similar (32.14 ± 1.83 min and 32.07 ± 1.50 min, respectively), reaching a mean speed of about 62 m/min in both groups. Similarly, plasma cortisol levels measured at the end of the study, which increased with exhaustion, did not change in hesperidin-supplemented rats (Figure 1c). Likewise, relative heart weight was higher in exhausted animals than in sedentary animals from both non-supplemented and supplemented groups. However, 24 h later, while heart weight was completely restored in non-supplemented animals, it tended to remain higher in the hesperidin-supplemented group (*p* = 0.09 between H-SED and H-TE24) (Figure 1d). 

### 3.2. Blood Leukocytes Count and Lymphocyte Proportions

**Figure 2:**
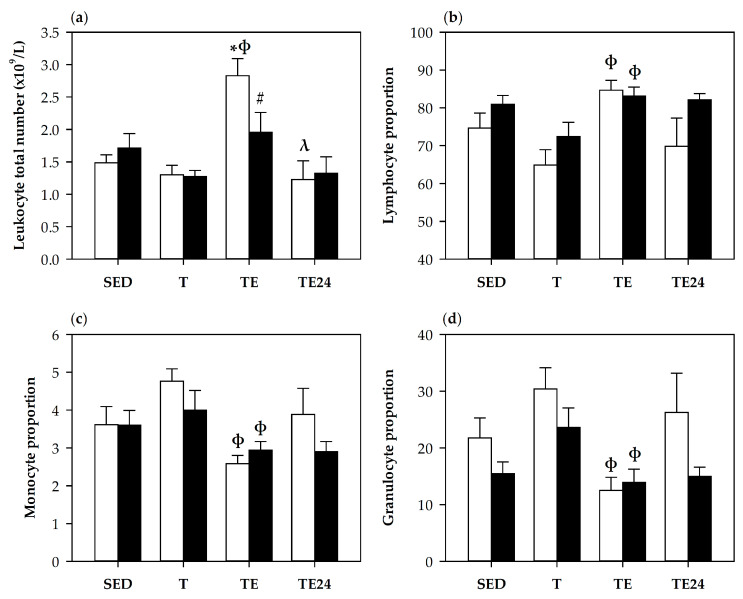
Blood leukocytes counts (**a**), and percentages of lymphocytes (**b**), monocytes (**c**) and granulocytes (**d**). The non-supplemented groups are represented by white bars and the hesperidin-supplemented groups by black bars. SED = sedentary groups; T = trained groups; TE = T groups with an additional exhaustion test; TE24 = TE groups 24 h after the exhaustion test. Data are expressed as mean ± SEM (*n* = 8). Statistical difference (Mann–Whitney *U* test): * *p* < 0.05 vs. SED group; ϕ *p* < 0.05 vs. T group; λ *p* < 0.05 vs. TE group; # significant differences between non-supplemented and the counterpart hesperidin-supplemented group (*p* < 0.05).

The counts of blood leukocytes and the proportion of lymphocytes, monocytes and granulocytes were assessed by an automatic hematology analyzer at the end of the study (Figure 2). Immediately after the final exhaustion test (TE group), blood leukocyte counts increased in non-supplemented rats (Figure 2a). This increase was prevented by hesperidin supplementation (H-TE group). The percentages of lymphocytes, granulocytes and monocytes (Figure 2b–d) showed that the leukocytosis observed in the TE group was mainly due to lymphocytes because its percentage increased and that of granulocytes and monocytes decreased with respect to the T group. 

**Figure 3:**
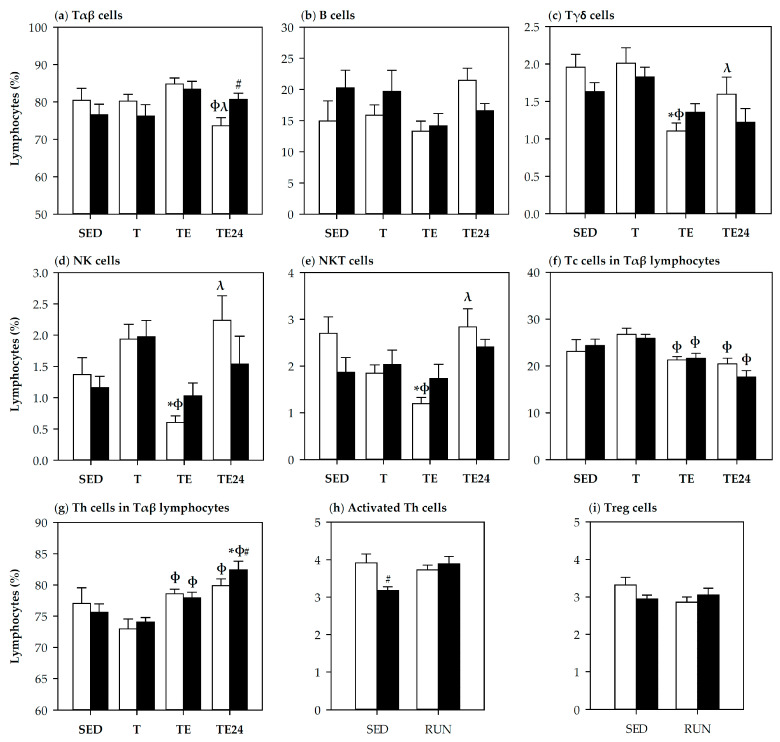
Percentages of blood lymphocytes: T (TCRαβ+) (**a**), B (TCRαβ- TCRγδ-CD161b-) (**b**), Tγδ (TCRγδ+) (**c**), natural killer (NK) (CD161b+) (**d**), natural killer T (NKT) (CD161b+TCRαβ+) (**e**), Th (CD4+CD161b- in TCRαβ+) (**f**), Tc (CD8+CD161b- in TCRαβ+) (**g**), Tact (CD25+Foxp3- in CD4+ TCRαβ+) (**h**), and Treg (CD25+Foxp3+ in CD4+ TCRαβ+) (**i**) cells. The proportions of Treg and Tact cells were established one week before final exhausting test (therefore, all runners). The non-supplemented groups are represented by white bars and the hesperidin-supplemented groups by black bars. SED = sedentary groups; T = trained groups; TE = T groups with an additional exhaustion test; TE24 = TE groups 24 h after the exhaustion test. Data are expressed as mean ± SEM (*n* = 8 for **a**–**g**, *n* = 8–15 for **h**,**i**). Statistical difference (Mann–Whitney *U* test): * *p* < 0.05 vs. SED group; ϕ *p* < 0.05 vs. T group; λ *p* < 0.05 vs. TE group; # significant differences between non-supplemented and the counterpart hesperidin-supplemented group (*p* < 0.05).

Flow cytometry analysis of blood lymphocytes allows the proportions of the main blood lymphocyte subsets to be established (Figure 3). Although trained rats (T group) did not significantly modify blood lymphocyte population distribution in comparison with the SED group, the final exhaustion test caused changes that were mainly observed when comparing with the T group. 

The lymphocytosis observed by hematology analyzer was due to a tendency to increase the T cell proportion in the TE group when compared with the T group (*p* = 0.1, TE group vs. T group). A similar tendency was observed in the exhausted rats receiving hesperidin (Figure 3a). When considering the Th and Tc cell subsets in T lymphocytes, the proportion of Th cells increased and that of Tc cells decreased in the TE group (*p* = 0.05 vs. T group), indicating that Th lymphocytes were mainly involved in the lymphocytosis induced by the exhausting exercise (Figure 3f–g). 

One day after the final exhaustion test, the non-supplemented animals (TE24 group) showed a decrease in the T lymphocyte percentage (*p* = 0.049 vs. T group, *p* = 0.009 vs. TE group). On the contrary, hesperidin supplementation kept a higher T cell proportion in this group (*p* = 0.049, H-TE24 group vs. TE24 group), mainly due to a higher percentage in the Th cell subset (*p* = 0.046, H-TE24 group vs. TE24 group).

Blood NK, NKT (NK with TCRαβ+) and TCRγδ+ cell proportions decreased immediately after exhaustion, nevertheless, hesperidin supplementation tended to attenuate the reduction of NK and NKT cells (*p* = 0.063 and *p* = 0.085 between the TE group and H-TE group, respectively). The percentage of TCRγδ+ cells did not change due to hesperidin supplementation (Figure 3c–e).

In addition, one week before the end of the study, the proportion of blood regulatory T cells (Treg) and activated Th cells (Tact) were evaluated (Figure 3h–i). The Treg percentage was not modified by exercise. However, the proportion of Tact cells in blood was lower in hesperidin-supplemented SED animals than in the non-supplemented counterpart group (*p* = 0.010). 

### 3.3. Lymphocyte Composition in Thymus and Spleen

Changes in thymus lymphocyte composition due to exhausting exercise and hesperidin supplementation were also assessed (Table 1). Just training did not significantly modify the thymocyte subsets studied here. Nevertheless, after running the final exhaustion test, some changes appeared when compared with trained rats. Non-supplemented animals showed a lower proportion of TCRαβ+ cells immediately after carrying out the additional final exhaustion test (TE group) than the other conditions (*p* = 0.050 vs. SED group, *p* = 0.022 vs. T group), which was accompanied by a tendency to decrease the percentage of CD4+CD8- cells (*p* = 0.064, TE group vs. T group) and to increase that of CD4-CD8+ cells (*p* = 0.064, TE group vs. T group). One day later, there was a notable increase in TCRαβ+ (*p* = 0.000, TE24 group vs. TE group) and CD4-CD8- (*p* = 0.049 vs. T group) cell proportions. These changes were also observed in hesperidin-supplemented groups. However, it is noteworthy that hesperidin supplementation decreased the relative proportion of CD4-CD8+ thymocytes in the H-TE group with respect to the TE group (*p* = 0.003) and showed a higher proportion of the CD4+CD8- subset 24 h later (*p* = 0.018, TE24 group vs. H-TE24 group).

Spleen lymphocyte composition was also studied (Table 2). Intensive training did not change the main lymphocyte subsets, i.e., B, T, Th and Tc cells, but did change the proportion of TCRγδ+ cells and NK cells, which decreased 24 h after the final exhaustion test (*p* = 0.004, TE24 group vs. SED group for TCRγδ+ cells; *p* = 0.05 vs. T group for NK cells). Hesperidin supplementation increased the proportion of T cells 24 h after the exhaustion test (*p* = 0.018, TE24 group vs. H-TE24 group).

### 3.4. Spleen Lymphocyte Proliferation

**Figure 4:**
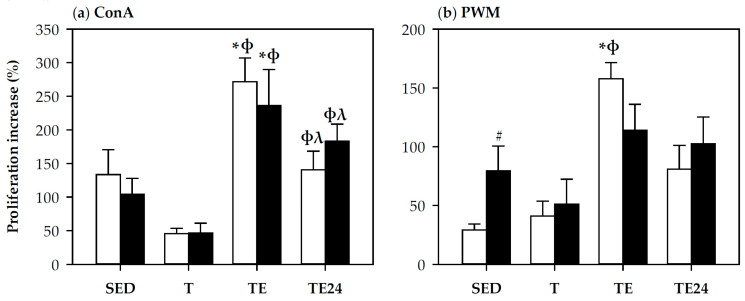
Proliferation capacity of spleen T lymphocytes (**a**) and B lymphocytes (**b**). The non-supplemented groups are represented by white bars and the hesperidin-supplemented groups by black bars. SED = sedentary groups; T = trained groups; TE = T groups with an additional exhaustion test; TE24 = TE groups 24 h after the exhaustion test. Data are expressed as mean ± SEM (*n* = 8). Statistical differences (Mann–Whitney *U* test for **a**, and two-way ANOVA followed by Tuckey for **b**): * *p* < 0.05 vs. SED group; ϕ *p* < 0.05 vs. T group; λ *p* < 0.05 vs. TE group; # significant differences between non-supplemented and the counterpart hesperidin-supplemented group (*p* < 0.05).

The functionality of spleen T and B cells was also assessed by their proliferation after ConA and PWM stimulations, respectively (Figure 4). The proliferation capacity of T cells was not modified by training but increased immediately after exhaustion (TE group) in both non-supplemented and hesperidin-supplemented animals (*p* < 0.05 vs. SED group and T group) (Figure 4a). One day after the final exhaustion test, T cell proliferation rate decreased in comparison with the TE group (*p* = 0.022, TE24 group vs. TE group) but remained higher than in the T group (*p* = 0.010, TE24 group vs. T group).

B cell proliferative capacity increased after exhaustion in non-supplemented animals (*p* = 0.009, TE group vs. SED group, *p* = 0.006, TE group vs. T group) (Figure 4b), nevertheless, hesperidin supplementation tended to attenuate this effect (*p* = 0.1, TE group vs. H-TE group). Moreover, hesperidin enhanced B cell proliferative ability in sedentary animals (*p* = 0.038, SED group vs. H-SED group).

### 3.5. NK Cell Cytotoxic Activity

**Figure 5:**
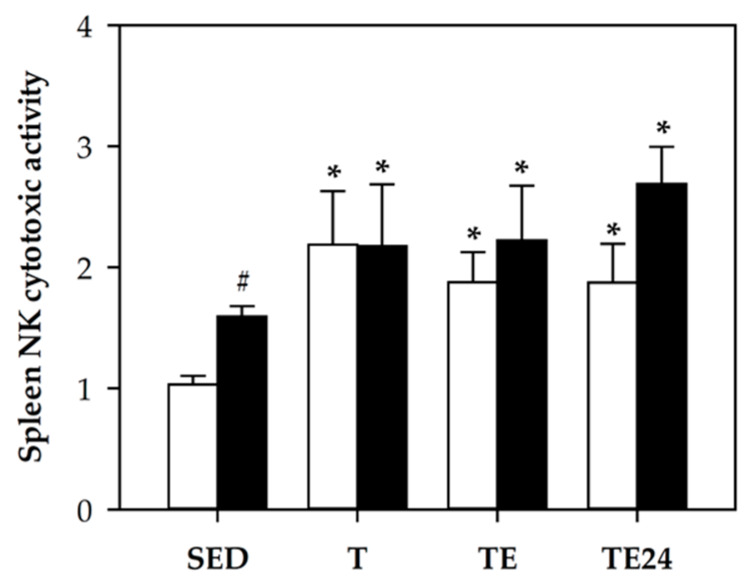
Spleen NK cytotoxic activity (number of dead target cells per 100 effector cells). The non-supplemented groups are represented by white bars and the hesperidin-supplemented groups by black bars. SED = sedentary groups; T = trained groups; TE = T groups with an additional exhaustion test; TE24 = TE groups 24 h after the exhaustion test. Data are expressed as mean ± SEM (*n* = 8). Statistical difference (Mann–Whitney *U* test): * *p* < 0.05 vs. SED group; # significant differences between non-supplemented and the counterpart hesperidin-supplemented group (*p* < 0.05).

The cytotoxic function of spleen NK cells was determined (Figure 5). In both non-supplemented and hesperidin-supplemented animals, there was a higher cytotoxic activity in all intensively trained and exhausted rats (*p* < 0.05 between SED groups and T, TE, and TE24 groups). Moreover, hesperidin increased the NK cytotoxic activity in sedentary animals (*p* = 0.000, SED group vs. H-SED group) and tended to raise that of the TE24 group (*p* = 0.09, TE24 group vs. H-TE24).

### 3.6. Phagocytic Activity

**Figure 6:**
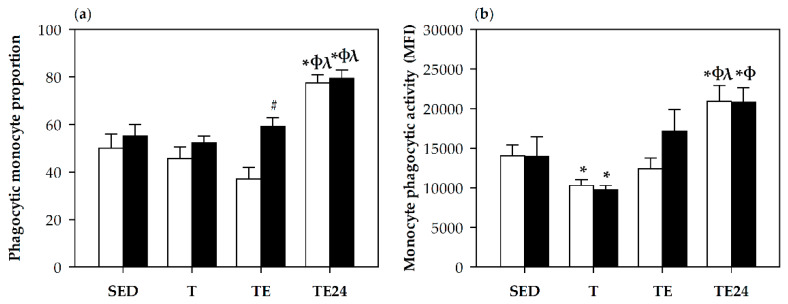
Phagocytic monocyte proportion (**a**) and monocyte phagocytic activity expressed as mean fluorescence intensity (MFI) (**b**). The non-supplemented groups are represented by white bars and the hesperidin-supplemented groups by black bars. SED = sedentary groups; T = trained groups; TE = T groups with an additional exhaustion test; TE24 = TE groups 24 h after the exhaustion test. Data are expressed as mean ± SEM (*n* = 7–8). Statistical difference (Mann–Whitney U test): * *p* < 0.05 vs. SED group; ϕ *p* < 0.05 vs. T group; λ *p* < 0.05 vs. TE group; # significant differences between non-supplemented and the counterpart hesperidin-supplemented group (*p* < 0.05).

The proportion of blood monocytes with phagocytic ability, as well as their phagocytic activity was assessed at the end of the study (Figure 6). The percentage of phagocytic monocytes was influenced both by intensive training and hesperidin supplementation (Figure 6a). In particular, 24 h after the final exhaustion test, the proportion of phagocytic monocytes increased (*p* = 0.004, TE24 group vs. SED, T and TE groups). This proportion was also enhanced by hesperidin supplementation immediately after carrying out the final exhaustion test (*p* = 0.006, TE group vs. H-TE group). Concerning the functionality of these monocytes, cells from the T group showed a lower phagocytic activity than those from the SED group (*p* = 0.037). Moreover, the increases in monocyte proportions 24 h after the exhaustion test were associated with a higher phagocytic activity in both hesperidin and non-hesperidin groups (*p* < 0.05, TE24 group vs. SED, T and TE groups) (Figure 6b).

No changes in the proportion of phagocytic granulocytes and their activity were observed due to training, exhaustion test or hesperidin supplementation (data not shown). 

### 3.7. Macrophage Cytokine Production

The production of IFN-γ, IL-1β, IL-6, TNF-α, and IL-10 was quantified in supernatants of LPS-stimulated peritoneal macrophages obtained from sedentary and runner rats supplemented or not with hesperidin (Table 3).

Concerning IFN-γ secretion, in non-supplemented rats, intensive training (T group) was accompanied by a higher IFN-γ secretion than SED animals (*p* = 0.014), which was prevented by the hesperidin supplementation (*p* = 0.05, H-T group vs. T group). On the other hand, hesperidin supplementation induced a higher IFN-γ production in sedentary animals (*p* = 0.042, SED group vs. H-SED group) and tended to increase its levels 24 h after the final exhaustion test (*p* = 0.076, TE24 group vs. H-TE24 group).

The secretion of IL-1β was not significantly modified by either intensive training or hesperidin supplementation.

IL-6 secretion increased by exercise, but there was only a significant effect just immediately after exhaustion (*p* = 0.018, SED group vs. TE group). Hesperidin-supplemented trained animals kept the IL-6 secretion closer to the values obtained in their sedentary counterpart animals (H-SED group).

TNF-α secretion increased immediately after exhaustion as well as 24 h later, when compared to the levels in trained rats (*p* = 0.05 and *p* = 0.048 T group vs. TE and TE24 groups, respectively). Hesperidin supplementation did not modify the TNF-α secretion profile.

IL-10 secretion was not altered by training or exhaustion but changed due to hesperidin supplementation. The levels of IL-10 in trained rats were lower in those animals receiving hesperidin than in those non-supplemented (*p* = 0.002, T group vs. H-T group).

## 4. Discussion

Previous studies demonstrated that rats submitted to intensive training and exhausting exercise exhibited alterations in both innate and acquired immunity [6,7]. In the current study we show the potential role of hesperidin supplementation in preventing some of these modifications.

First of all, as already reported [22], hesperidin supplementation partially improved exercise performance throughout the continuous training, avoiding the decrease in running capacity observed in the non-supplemented animals on Monday after resting for the weekend. Nevertheless, this effect was not observed in the exhaustion tests from the last three Fridays, and the additional final exhausting test, hence both non-supplemented and hesperidin-supplemented groups evidenced a decline in physical performance probably due to the high frequency of training and the lack of an adequate recovery between training sessions. The performance increase by hesperidin observed in the first weeks agrees with those reported in the cycling time-trial performance in trained male athletes after the intake of a citrus flavonoid extract [20], and those observed in healthy male amateur cyclists who carried out a repeated sprint test on a cycle ergometer after the acute intake of 500 mg of 2S-Hesperidin (Cardiose^®^) [25]. This increase in performance could be related to the antioxidant effect of hesperidin. In this context, rats receiving hesperidin and submitted to swimming or running, improved the antioxidant biomarkers and exercise performance [21,22]. Nevertheless, it has been highlighted that exercise-induced oxidative stress has been postulated as a positive regulator of the adaptation of endurance training which could explain that at the end of the study hesperidin supplementation did not produce a better performance [26]. Moreover, the lack of effect at the end of the study could be due to hesperidin inhibitory effects on prostaglandin synthesis [27,28]. In this context, the decrease of prostaglandin synthesis by anti-inflammatory drugs has been associated with an inhibition of strength and muscle hypertrophic adaptations [29,30]. In any case, it seems that hesperidin supplementation could be useful in increasing exercise performance in some cases and further studies should be carried out to establish the optimal dosage for these effects.

Considering the innate immunity, we have previously reported that a similar exhausting exercise reduced the spleen NK cell proportion but increased its cytotoxic activity, decreased the phagocytic activity by blood phagocytes, and altered the pattern of peritoneal macrophage cytokine secretion [6]. In the current study, the final exhaustion test decreased the blood percentage of the minor subsets NK, NKT and TCRγδ+ cells, and 24 h later decreased the proportion of NK cells in the spleen. Nevertheless, training and exhaustion were accompanied by an increase in cytotoxic activity, which agrees with previous results reported about NK cell proportion and activity [6,7,31]. Herein hesperidin supplementation tended to avoid the decrease in blood NK and NKT cell proportion and was able to increase the cytotoxic activity of NK cells, although this effect only reached statistical significance in sedentary animals. Although these results must be confirmed with further experiments, they could suggest a protective effect of hesperidin in NK cell function involved in the innate immune response and modified by intensive training. In this regard, an in vitro study shows that hesperidin increased spleen NK cell function, as well as the activity of splenic cytotoxic T lymphocytes [32]. Moreover, it has been described that daily anthocyanin consumption for 6 weeks prevented the decrease in blood NK cell count induced by 2.5 h of running by well-trained subjects [33]. Similarly, the enhancing effect of polyphenols, such as myricetin and resveratrol, among others, on NK cell activity has been reported [34,35,36]. Nevertheless, a study including the daily intake of 292 mg of hesperidin did not change blood NK cell proportion or their cytotoxic activity in healthy well-nourished humans [37]. Although there is some evidence, further studies focused on the effect of hesperidin on NK activity when there is a viral infection after intensive training, where these cells have a defensive role, must shed some light on the effect of this flavanone on NK cells. In this context, hesperidin has been considered as a potential protective agent against various infectious factors [38].

With regard to phagocytosis, our results showed that the proportion of blood phagocytic monocytes increased 24 h after exhaustion as did their phagocytic activity, in agreement with previous studies [6,39]. The hesperidin supplementation increased the proportion of blood phagocytic monocytes, which was more evident immediately after exhaustion, in consonance with other studies that have suggested the helpful effect of this flavanone in restoring to normal levels the phagocytic index of neutrophils in Wistar rats after a toxic insult administration [40], and in increasing in vitro lysosomal phosphatase activity of macrophages, which may correlate to degranulation in phagocytosis [32]. Here, this enhancing effect could help to counteract some of the immediate immunodepressant effects of exhaustion, as phagocytosis is a key process in fighting against potential pathogenic invaders.

Intensive training and exhaustion also affect cytokine secretion by LPS-stimulated peritoneal macrophages. Previous studies demonstrate attenuations in the pro-inflammatory cytokine secretion, such as TNF-α and IL-12, with intensive training, which were overcome after exhaustion [6]. Here, we found no significant effects on the levels of TNF-α and IL-1β after training but there was an increase in TNF-α secretion after exhaustion, thus evidencing an inflammatory status induced by this high-load exercise. Moreover, there was an increase in IL-6 secretion by exhaustion, as previously reported in trained and exhausted rats [6] and also reported in plasma due to the secretion of muscle fibers [41,42,43]. In this case, hesperidin did not play a significant preventive effect. This agrees with a study reporting that daily hesperidin supplementation (100 mg/kg BW) did not restore the higher IL-6 secretion induced by high-fat diet intake in Wistar rats [44]. Nevertheless, other studies show that crude polyphenols extracted from blossoms of *Citrus aurantium* L. var. *amara* Engl., including neohesperidin and hesperitin, displayed an inhibitory effect on secretion of IL-6, TNF-α and IL-1β by murine macrophage RAW264.7 cell line in a concentration-dependent manner [45]. On the one hand, the secretion of IFN-γ increased remarkably with intensive training in peritoneal macrophages from trained rats and, interestingly, hesperidin supplementation prevented this effect in accordance with the inhibitory effect of hesperidin on IFN-γ previously described in an in vitro study using human mesenchymal stem cells [46] and in a mouse model of fulminant hepatitis [47]. However, in sedentary conditions, also 24 h after exhaustion, hesperidin increased (or tended to increase) the secretion of IFN-γ by peritoneal macrophages, suggesting its enhancing effect on innate immunity. Finally, in trained rats, hesperidin supplementation decreased the secretion of IL-10, a typical cytokine from M2 macrophages. It has been reported that polyphenols tend to polarize macrophages towards the M2 phenotype due to their anti-inflammatory properties [48]. However, in the current study, after performing an exhausting exercise the immune function is impaired, and hesperidin, as immunomodulator, may enhance macrophages’ pro-inflammatory functions in order to decrease the risk of potential infectious diseases. Our results partially agree with those reported by Dourado et al. [49], showing a lower secretion of IL-10 by LPS-stimulated macrophages from non-exercised mice treated orally with hesperidin for 2 weeks, and also by in vitro studies in mesenchymal stem cells [46]. However they disagree with another in vitro study showing a higher expression of IL-10 in the RAW264.7 cell line after hesperidin incubation [50]. Thus, although the effect of polyphenols in general, and hesperidin in particular, in some macrophage functions such as production of reactive species of nitrogen and oxygen have been widely studied [22,48], the impact of hesperidin in macrophage phagocytosis, cytokine secretion and phenotype polarization in both healthy and immunosuppressed conditions remains controversial and must be elucidated in future studies.

Besides the effects of exercise and hesperidin in the innate immunity, some approaches in acquired immunity have been carried out. As observed in previous studies [6], an exhausting test in intensively trained animals induced an immediate leukocytosis that was overcome 24 h later. The leukocytosis was due to an increase in the lymphocyte counts. This fact has been associated with the increase of adrenalin secretion by exhaustion, and its ability to release leukocytes from the marginal compartment [51]. The analysis of blood lymphocytes by flow cytometry revealed that exhaustion was accompanied by an increase in Th cell proportion, as previously reported [7], and is in agreement with the fact that T cells mobilize faster than other lymphocytes from the spleen compartment [52]. Interestingly, hesperidin supplementation prevented the leukocytosis in exhausted rats. This could be due to an attenuating effect on the catecholamine release, although hesperidin was not able to decrease the cortisol hormone released by exhaustion as observed here. In addition, hesperidin attenuated the proportion of blood T-activated lymphocytes in sedentary animals, which could be a reflection of its modulatory effect on the acquired immunity. Nevertheless, previous studies performed in healthy humans showed that hesperidin consumption induced no changes in T lymphocyte activation [37].

When considering the thymus, we observed that exhaustion reduced the proportion of the mature cells (TCRαβ+ cells and showed a tendency to decrease the CD4+CD8- subset), probably indicating the mobilization of these cells to the blood. Such decrease in TCRαβ+ cells was not prevented by hesperidin but, interestingly, its supplementation increased the CD4+CD8- proportion 24 h after exhaustion. These effects would suggest an enhanced effect of the flavanone on Th cell maturation. On the other hand, spleen T (Th and Tc) and B lymphocyte proportions were not modified by either intensive training or the final exhaustion test. Nevertheless, the administration of hesperidin was able to increase the relative proportion of T cells as significantly observed 24 h after the final exhaustion test (and with a tendency in the sedentary group). Although they do not match with those observed in blood, the results agree with the increase in CD4+CD8- thymocytes and also with results reported in mesenteric lymph nodes [18,19], which could be due to a more intense effect of hesperidin on lymphoid tissues.

Not only was lymphocyte composition modified by exhausting exercise, but also their functionality as it was quantified by means of their proliferation capacity. As previously reported [7], the proliferation of spleen T and B lymphocytes increased immediately after exhaustion. Moreover 24 h later, there was an increase in NK cytotoxicity and monocyte phagocytic activity, which partially disagrees with recent publications claiming that exercise may induce only a transient redistribution of immune cells at 1–2 h following a bout of intensive training, returning to baseline within 24 h [10]. On the other hand, in sedentary animals, we found a higher B cell proliferation capacity and no changes in that of T cells due to hesperidin supplementation. Nevertheless, we did not observe a marked protective effect of hesperidin on exhaustion-induced cell proliferation changes, only a tendency to normalize that of B cells. Although these results must be confirmed with future experiments, they would partially agree with previous studies that showed an increase in both T and B cell proliferation of murine splenocytes after 24 h in vitro stimulation with hesperidin, both in the absence of mitogen and after 48 h LPS or lectin-stimulation [32]. However, there is also evidence of the antiproliferative properties of some polyphenols in cancer [53] and, therefore, further studies would be necessary to elucidate the controversial effects of these bioactive compounds on cell proliferation in both overactivated and suppressed immune system conditions.

## 5. Conclusions

In summary, intensive training and an additional exhaustion test modify both innate and acquired immunity in rats. Here we demonstrate that the administration of hesperidin during training prevents the decrease in performance after a two-day resting period and enhances some aspects of the immune response. In some conditions, hesperidin is able to enhance the cytotoxic function of NK cells as well as the proportion of phagocytic cells, whereas it has an attenuating effect on some cytokines secreted by macrophages. Moreover, hesperidin prevents the leukocytosis induced by exhaustion and promotes the abundance of T (Th) cells in the thymus, blood and spleen, which can be observed, above all, the day after the exhaustion test. These immunoenhancing effects of this flavanone in intensive exercise may be confirmed and completed with further experiments. Such experiments should be focused on ascertaining whether the induction of an infectious process after training and exhaustion can be attenuated by the hesperidin administration.

## Figures and Tables

**Table 1:** Percentages of thymus lymphocytes: TCRαβ+; double negative (DN) (CD4-CD8-); double positive (DP) (CD4+CD8+); mature TCD4+ (CD4+CD8-); and mature TCD8+ (CD4-CD8+) cells.

Thymus Lymphocyte Composition					
	Hesperidin	SED	T	TE	TE24
% in total lymphocytes:					
TCRαβ+	-	12.35 ± 1.27	14.94 ± 1.34	10.19 ± 0.74 ^ϕ^	15.76 ± 1.13 ^λ^
	+	15.11 ± 1.42	12.72 ± 0.64	9.23 ± 0.98	17.31 ± 1.42 ^λ^
DN	-	3.62 ± 0.69	3.15 ± 0.45	2.82 ± 0.11	4.48 ± 0.43 ^λ^
	+	3.90 ± 0.52	3.15 ± 0.35	2.24 ± 0.31	4.44 ± 0.38 ^λ^
DP	-	81.85 ± 2.59	81.71 ± 1.88	84.11 ± 0.76	80.46 ± 1.48
	+	81.71 ± 1.06	84.51 ± 1.06	86.73 ± 1.59	76.5 ± 2.21
TCD4+	-	8.90 ± 1.47	8.93 ± 0.91	6.62 ± 0.37	8.21 ± 0.81
	+	10.36 ± 0.82	7.99 ± 0.71	5.93 ± 1.34	12.57 ± 0.98
TCD8+	-	5.62 ± 0.51	4.61 ± 0.61	6.25 ± 0.29	5.86 ± 0.39
	+	4.53 ± 0.41	4.33 ± 0.32	4.11 ± 0.19 ^#^	6.16 ± 0.37

SED = sedentary groups; T = trained groups; TE = T groups with an additional exhaustion test; TE24 = TE groups 24 h after the exhaustion test. - = non-supplemented groups; + = hesperidin-supplemented groups. Data are expressed as mean ± SEM (*n* = 8). Statistical difference (Mann–Whitney *U* test): ϕ *p* < 0.05 vs. T group; λ *p* < 0.05 vs. TE group; ^#^ significant differences between non-supplemented and the counterpart hesperidin-supplemented group (*p* < 0.05).

**Table 2:** Percentages of spleen lymphocytes: T (TCRαβ+); B (CD45RA+); Tγδ (TCRγδ+); NK (CD161b+); NKT (CD161b+ TCRαβ+); Th (CD4+CD161b- in TCRαβ+); and Tc (CD8+CD161b- in TCRαβ+) cells.

Spleen Lymphocyte Composition					
	Hesperidin	SED	T	TE	TE24
**% in total lymphocytes:**					
T	-	41.68 ± 1.72	40.41 ± 5.49	43.71 ± 2.68	43.24 ± 1.84
	+	47.07 ± 2.13	45.47 ± 1.89	43.51 ± 2.64	50.91 ± 1.16 ^#^
B	-	36.83 ± 1.63	32.06 ± 2.06	38.10 ± 2.20	37.24 ± 1.97
	+	33.35 ± 1.96	34.46 ± 2.18	37.60 ± 1.62	32.41 ± 1.21
Tγδ	-	3.95 ± 0.22	3.32 ± 0.48	3.54 ± 0.26	2.89 ± 0.20 *
	+	3.64 ± 0.24	3.24 ± 0.24	3.44 ± 0.12	2.78 ± 0.22 *
NK	-	7.92 ± 1.05	6.21 ± 0.90	5.28 ± 0.51	5.01 ± 0.80 ^ϕ^
	+	6.43 ± 0.41	6.51± 1.13	5.70 ± 0.84	4.10 ± 0.41 ^ϕ^
NKT	-	4.71 ± 0.80	3.29 ± 0.49	3.48 ± 0.22	3.21 ± 0.29
	+	3.50 ± 0.17	4.24 ± 0.67	3.10 ± 0.23	3.33 ± 0.30
**% in T lymphocytes:**					
Th	-	74.88 ± 0.57	72.19 ± 1.09	74.09 ± 0.74	74.01 ± 0.92
	+	72.59 ± 1.66	70.00 ± 3.26	74.43 ± 0.38	75.34 ± 0.46
Tc	-	27.87 ± 1.27	28.57 ± 0.70	27.23 ± 0.85	26.60 ± 0.74
	+	28.21 ± 1.95	30.36 ± 2.73	27.71 ± 0.47	26.95 ± 1.90

SED = sedentary groups, T = trained groups, TE = T groups with an additional exhaustion test, TE24 = TE groups 24 h after the exhaustion test. - = non-supplemented groups; + = hesperidin-supplemented groups. Data are expressed as mean ± SEM (*n* = 8). Statistical difference (Mann-Whitney *U* test): * *p* < 0.05 vs. SED group; ^ϕ^ *p* < 0.05 vs. T group; ^#^ significant differences between non-supplemented and the counterpart hesperidin-supplemented group (*p* < 0.05).

**Table 3:** Cytokine concentration (pg/mL) released by peritoneal macrophages stimulated by LPS.

Peritoneal Macrophage Cytokine Secretion					
	Hesperidin	SED	T	TE	TE24
IFN-γ	-	28.14 ± 10.33	161.36 ± 34.75 *	46.01 ± 8.64 ^ϕ^	34.92 ± 15.00 ^ϕ^
	+	63.12 ± 11.19 ^#^	46.92 ± 19.32 ^#^	33.48 ± 10.40	124.6 ± 55.87
IL-1β	-	131.7 ± 23.01	102.7 ± 23.15	165.3 ± 12.05	141.7 ± 31.66
	+	127.6 ± 18.21	90.29 ± 16.41	147.1 ± 37.84	168.8 ± 21.69
IL-6	-	710.6 ± 167.3	1282.5 ± 155.4	1635.0 ± 224.6 *	1080.9 ± 316.5
	+	1054.0 ± 192.2	1032.2 ± 222.5	1358.7 ± 334.9	1737.2 ± 268.3
TNF-α	-	304.6 ± 76.60	146.8 ± 38.30	408.5 ± 63.30 ^ϕ^	359.2 ± 86.57 ^ϕ^
	+	350.0 ± 54.97	238.9 ± 56.38	353.9 ± 73.59	419.0 ± 51.32
IL-10	-	83.41 ± 19.73	97.22 ± 16.57	58.02 ± 28.21	87.03 ± 17.78
	+	118.7 ± 27.28	47.40 ± 8.74 ^#^	54.48 ± 15.14	76.70 ± 27.53

SED = sedentary groups; T = trained groups; TE = T groups with an additional exhaustion test; TE24 = TE groups 24 h after the exhaustion test. – = non-supplemented groups; + = hesperidin-supplemented groups. Data are expressed as mean ± SEM (*n* = 5–8). Statistical difference (Mann–Whitney *U* test): * *p* < 0.05 vs. SED group; ^ϕ^ *p* < 0.05 vs. T group; ^#^ significant differences between non-supplemented and the counterpart hesperidin-supplemented group (*p* < 0.05).

## References

[1] Warburton D.E.R., Nicol C.W., Bredin S.S.D. (2006). Health benefits of physical activity: The evidence. Can. Med. Assoc. J..

[2] Miles L. (2007). Physical activity and health. Br. Nutr. Found..

[3] Pape K., Ryttergaard L., Rotevatn T.A., Nielsen B.J., Torp-Pedersen C., Overgaard C., BØggild H. (2016). Leisure-time physical activity and the risk of suspected bacterial infections. Med. Sci. Sports Exerc..

[4] Walsh N.P., Gleeson M., Shephard R.J., Gleeson M., Woods J.A., Bishop N.C., Fleshner M., Green C., Pedersen B.K., Hoffman-Goetz L. (2011). Position statement part one: Immune function and exercise. Exerc. Immunol. Rev..

[5] Walsh N.P., Gleeson M., Pyne D.B., Nieman D.C., Dhabhar S., Shephard R.J., Oliver S.J., Bermon S., Kajeniene A. (2011). Position statement part two: Maintaining immune health. Exerc. Immunol. Rev..

[6] Estruel-Amades S., Camps-Bossacoma M., Massot-Cladera M., Pérez-Cano F.J., Castell M. (2020). Alterations in the innate immune system due to exhausting exercise in intensively trained rats. Sci. Rep..

[7] Estruel-Amades S., Ruiz-Iglesias P., Périz M., Franch À., Pérez-Cano F.J., Camps-Bossacoma M., Castell M. (2019). Changes in lymphocyte composition and functionality after intensive training and exhausting exercise in rats. Front. Physiol..

[8] Schwellnus M., Soligard T., Alonso J.-M., Bahr R., Clarsen B., Dijkstra H.P., Gabbett T.J., Gleeson M., Hägglund M., Hutchinson M.R. (2016). How much is too much? (Part 2) International olympic committee consensus statement on load in sport and risk of illness. Br. J. Sports Med..

[9] Cantó E., Roca E., Perea L., Rodrigo-Troyano A., Suarez-Cuartin G., Giner J., Feliu A., Soria J.M., Nescolarde L., Vidal S. (2018). Salivary immunity and lower respiratory tract infections in non-elite marathon runners. PLoS ONE.

[10] Campbell J.P., Turner J.E. (2018). Debunking the myth of exercise-induced immune suppression: Redefining the impact of exercise on immunological health across the lifespan. Front. Immunol..

[11] Kawamura T., Muraoka I. (2018). Exercise-induced oxidative stress and the effects of antioxidant intake from a physiological viewpoint. Antioxidants.

[12] Nieman D., Mitmesser S. (2017). Potential impact of nutrition on immune system recovery from heavy exertion: A metabolomics perspective. Nutrients.

[13] De Ferrars R.M., Czank C., Zhang Q., Botting N.P., Kroon P.A., Cassidy A., Kay C.D. (2014). The pharmacokinetics of anthocyanins and their metabolites in humans. Br. J. Pharmacol..

[14] Davis J.M., Carlstedt C.J., Chen S., Carmichael M.D., Murphy E.A. (2010). The dietary flavonoid quercetin increases VO(2max) and endurance capacity. Int. J. Sport Nutr. Exerc. Metab..

[15] Nieman D.C., Williams A.S., Shanely R.A., Jin F., McAnulty S.R., Triplett N.T., Austin M.D., Henson D.A. (2010). Quercetin’s influence on exercise performance and muscle mitochondrial biogenesis. Med. Sci. Sports Exerc..

[16] Bowtell J.L., Sumners D.P., Dyer A., Fox P., Mileva K.N. (2011). Montmorency cherry juice reduces muscle damage caused by intensive strength exercise. Med. Sci. Sports Exerc..

[17] Parhiz H., Roohbakhsh A., Soltani F., Rezaee R., Iranshahi M. (2015). Antioxidant and anti-inflammatory properties of the citrus flavonoids hesperidin and hesperetin: An updated review of their molecular mechanisms and experimental models. Phyther. Res..

[18] Camps-Bossacoma M., Franch À., Pérez-Cano F.J., Castell M. (2017). Influence of hesperidin on the systemic and intestinal rat immune response. Nutrients.

[19] Estruel-Amades S., Massot-Cladera M., Pérez-Cano F.J., Franch À., Castell M., Camps-Bossacoma M. (2019). Hesperidin effects on gut microbiota and gut-associated lymphoid tissue in healthy rats. Nutrients.

[20] Overdevest E., Wouters J.A., Wolfs K.H.M., van Leeuwen J.J.M., Possemiers S. (2018). Citrus flavonoid supplementation improves exercise performance in trained athletes. J. Sports Sci. Med..

[21] De Oliveira D.M., Dourado G.K.Z.S., Cesar T.B. (2013). Hesperidin associated with continuous and interval swimming improved biochemical and oxidative biomarkers in rats. J. Int. Soc. Sports Nutr..

[22] Estruel-Amades S., Massot-Cladera M., Garcia-Cerdà P., Pérez-Cano F.J., Franch Á., Castell M., Camps-Bossacoma M. (2019). Protective effect of hesperidin on the oxidative stress induced by an exhausting exercise in intensively trained rats. Nutrients.

[23] Camps-Bossacoma M., Abril-Gil M., Saldaña-Ruiz S., Franch À., Pérez-Cano F.J., Castell M. (2016). Cocoa diet prevents antibody synthesis and modifies lymph node composition and functionality in a rat oral sensitization model. Nutrients.

[24] Grases-Pintó B., Abril-Gil M., Rodríguez-Lagunas M.J., Castell M., Pérez-Cano F.J., Franch À. (2018). Leptin and adiponectin supplementation modifies mesenteric lymph node lymphocyte composition and functionality in suckling rats. Br. J. Nutr..

[25] Martínez-Noguera F.J., Marín-Pagán C., Carlos-Vivas J., Rubio-Arias J.A., Alcaraz P.E. (2019). Acute effects of hesperidin in oxidant/antioxidant state markers and performance in amateur cyclists. Nutrients.

[26] Margaritelis N.V., Theodorou A.A., Paschalis V., Veskoukis A.S., Dipla K., Zafeiridis A., Panayiotou G., Vrabas I.S., Kyparos A., Nikolaidis M.G. (2018). Adaptations to endurance training depend on exercise-induced oxidative stress: Exploiting redox interindividual variability. Acta Physiol..

[27] Siddiqi A., Saidullah B., Sultana S. (2018). Anti-carcinogenic effect of hesperidin against renal cell carcinoma by targeting COX-2/PGE2 pathway in Wistar rats. Environ. Toxicol..

[28] Fu Z., Chen Z., Xie Q., Lei H., Xiang S. (2018). Hesperidin protects against IL-1β-induced inflammation in human osteoarthritis chondrocytes. Exp. Ther. Med..

[29] Markworth J.F., Vella L.D., Figueiredo V.C., Cameron-Smith D. (2014). Ibuprofen treatment blunts early translational signaling responses in human skeletal muscle following resistance exercise. J. Appl. Physiol..

[30] Lilja M., Mandić M., Apró W., Melin M., Olsson K., Rosenborg S., Gustafsson T., Lundberg T.R. (2018). High doses of anti-inflammatory drugs compromise muscle strength and hypertrophic adaptations to resistance training in young adults. Acta Physiol..

[31] Blank S.E., Johansson J.O., Pfister L.J., Gallucci R.M., Lee E.G., Meadows G.G. (1994). Mechanistic differences in NK cell cytolytic activity in treadmill-trained and chronic ethanol-consuming mice. J. Appl Physiol..

[32] Sassi A., Mokdad Bzéouich I., Mustapha N., Maatouk M., Ghedira K., Chekir-Ghedira L. (2017). Immunomodulatory potential of hesperetin and chrysin through the cellular and humoral response. Eur. J. Pharmacol..

[33] McAnulty L.S., Nieman D.C., Dumke C.L., Shooter L.A., Henson D.A., Utter A.C., Milne G., McAnulty S.R. (2011). Effect of blueberry ingestion on natural killer cell counts, oxidative stress, and inflammation prior to and after 2.5 h of running. Appl. Physiol. Nutr. Metab..

[34] Burkard M., Leischner C., Lauer U.M., Busch C., Venturelli S., Frank J. (2017). Dietary flavonoids and modulation of natural killer cells: Implications in malignant and viral diseases. J. Nutr. Biochem..

[35] Lindqvist C., Bobrowska-Hägerstrand M., Mrówczyńska L., Engblom C., Hägerstrand H. (2014). Potentiation of natural killer cell activity with Myricetin. Anticancer Res..

[36] Leischner C., Burkard M., Pfeiffer M.M., Lauer U.M., Busch C., Venturelli S. (2016). Nutritional immunology: Function of natural killer cells and their modulation by resveratrol for cancer prevention and treatment. Nutr. J..

[37] Perche O., Vergnaud-Gauduchon J., Morand C., Dubray C., Mazur A., Vasson M.P. (2014). Orange juice and its major polyphenol hesperidin consumption do not induce immunomodulation in healthy well-nourished humans. Clin. Nutr..

[38] Iranshahi M., Rezaee R., Parhiz H., Roohbakhsh A., Soltani F. (2015). Protective effects of flavonoids against microbes and toxins: The cases of hesperidin and hesperetin. Life Sci..

[39] Su S., Chen H., Jen C.J. (2001). Severe exercise enhances phagocytosis by murine bronchoalveolar macrophages. J. Leukoc. Biol..

[40] Hassouna I., Ibrahim H., Abdel Gaffar F., El-Elaimy I., Abdel Latif H. (2015). Simultaneous administration of hesperidin or garlic oil modulates diazinon-induced hemato- and immunotoxicity in rats. Immunopharmacol. Immunotoxicol..

[41] Pedersen B.K., Steensberg A., Schjerling P. (2001). Muscle-derived interleukin-6: Possible biological effects. J. Physiol..

[42] Sahl R.E., Andersen P.R., Gronbaek K., Morville T.H., Rosenkilde M., Rasmusen H.K., Poulsen S.S., Prats C., Dela F., Helge J.W. (2017). Repeated excessive exercise attenuates the anti-inflammatory effects of exercise in older men. Front. Physiol..

[43] Pedersen B.K., Steensberg A., Schjerling P. (2001). Exercise and interleukin-6. Curr. Opin. Hematol..

[44] Kumar R., Akhtar F., Rizvi S.I. (2020). Hesperidin attenuates altered redox homeostasis in an experimental hyperlipidaemic model of rat. Clin. Exp. Pharmacol. Physiol..

[45] Shen C.Y., Jiang J.G., Huang C.L., Zhu W., Zheng C.Y. (2017). Polyphenols from blossoms of Citrus aurantium L. var. amara Engl. show significant anti-complement and anti-inflammatory effects. J. Agric. Food Chem..

[46] Xiao S., Liu W., Bi J., Liu S., Zhao H., Gong N., Xing D., Gao H., Gong M. (2018). Anti-inflammatory effect of hesperidin enhances chondrogenesis of human mesenchymal stem cells for cartilage tissue repair. J. Inflamm..

[47] Bai X., Yang P., Zhou Q., Cai B., Buist-Homan M., Cheng H., Jiang J., Shen D., Li L., Luo X. (2017). The protective effect of the natural compound hesperetin against fulminant hepatitis in vivo and in vitro. Br. J. Pharmacol..

[48] Da Cunha L.R., Muniz-Junqueira M.I., Dos Santos Borges T.K. (2019). Impact of polyphenols in phagocyte functions. J. Inflamm. Res..

[49] Dourado G.K.Z.S., Ribeiro L.C.D.A., Carlos I.Z., César T.B. (2014). Orange juice and hesperidin promote differential innate immune response in macrophages ex vivo. Int. J. Vitam. Nutr. Res..

[50] Kong L.N., Lin X., Huang C., Ma T.T., Meng X.M., Hu C.J., Wang Q.Q., Liu Y.H., Shi Q.P., Li J. (2019). Hesperetin derivative-12 (HDND-12) regulates macrophage polarization by modulating JAK2/STAT3 signaling pathway. Chin. J. Nat. Med..

[51] Shephard R.J. (2003). Adhesion molecules, catecholamines and leucocyte redistribution during and following exercise. Sports Med..

[52] Krüger K., Mooren F.C. (2007). T cell homing and exercise. Exerc. Immunol. Rev..

[53] Jaganathan S.K., Mandal M. (2009). Antiproliferative effects of honey and of its polyphenols: A review. J. Biomed. Biotechnol..

